# Integration of Transcriptome and Methylome Analyses Provides Insight Into the Pathway of Floral Scent Biosynthesis in *Prunus mume*

**DOI:** 10.3389/fgene.2021.779557

**Published:** 2021-12-15

**Authors:** Xi Yuan, Kaifeng Ma, Man Zhang, Jia Wang, Qixiang Zhang

**Affiliations:** Beijing Key Laboratory of Ornamental Plants Germplasm Innovation and Molecular Breeding, National Engineering Research Center for Floriculture, Beijing Laboratory of Urban and Rural Ecological Environment, Engineering Research Center of Landscape Environment of Ministry of Education, Key Laboratory of Genetics and Breeding in Forest Trees and Ornamental Plants of Ministry of Education, School of Landscape Architecture, Beijing Forestry University, Beijing, China

**Keywords:** floral scent, phenylpropane biosynthesis pathway, Prunus mume, cytosine methylation, transcriptome

## Abstract

DNA methylation is a common epigenetic modification involved in regulating many biological processes. However, the epigenetic mechanisms involved in the formation of floral scent have rarely been reported within a famous traditional ornamental plant *Prunus mume* emitting pleasant fragrance in China. By combining whole-genome bisulfite sequencing and RNA-seq, we determined the global change in DNA methylation and expression levels of genes involved in the biosynthesis of floral scent in four different flowering stages of *P. mume*. During flowering, the methylation status in the “CHH” sequence context (with H representing A, T, or C) in the promoter regions of genes showed the most significant change. Enrichment analysis showed that the differentially methylated genes (DMGs) were widely involved in eight pathways known to be related to floral scent biosynthesis. As the key biosynthesis pathway of the dominant volatile fragrance of *P. mume*, the phenylpropane biosynthesis pathway contained the most differentially expressed genes (DEGs) and DMGs. We detected 97 DMGs participated in the most biosynthetic steps of the phenylpropane biosynthesis pathway. Furthermore, among the previously identified genes encoding key enzymes in the biosynthesis of the floral scent of *P. mume*, 47 candidate genes showed an expression pattern matching the release of floral fragrances and 22 of them were differentially methylated during flowering. Some of these DMGs may or have already been proven to play an important role in biosynthesis of the key floral scent components of *P. mume*, such as *PmCFAT1a*/*1c*, *PmBEAT36*/*37*, *PmPAL2*, *PmPAAS3*, *PmBAR8*/*9*/*10*, and *PmCNL1*/*3*/*5*/*6*/*14*/*17*/*20*. In conclusion, our results for the first time revealed that DNA methylation is widely involved in the biosynthesis of floral scent and may play critical roles in regulating the floral scent biosynthesis of *P. mume*. This study provided insights into floral scent metabolism for molecular breeding.

## Introduction

Floral fragrance is one of the most important ornamental traits of horticultural plants (Schiestl, 2010). More than 1700 floral scent substances have been identified, which are mainly biosynthesized through the terpenoid, phenylpropane, and fatty acid biosynthesis pathways. For example, *Cananga odorata* (Jin et al., 2015), *Magnolia champaca* (Dhandapani et al., 2017), and *Syringa oblata* (Zheng et al., 2015) have been found to have terpenoids as their main volatiles; *Petunia hybrida* and Damask rose (Karami et al., 2015) have phenylpropanes; and *Hedychium coronarium* (Yue et al., 2015) and *Rosa hybrida* have terpenoids and phenylpropanes. The diverse combinations of floral volatiles emitted confer the unique floral fragrances among different aromatic plants (Knudsen et al., 2006; Muhlemann et al., 2014). Genes related to the biosynthesis of substances conferring floral aromas have been comprehensively studied in model plants (Tzin and Galili, 2010), such as *Clarkia breweri*, *Antirrhinum majus*, *Rosa ssp*., and *Petunia hybrida* (Boatright et al., 2004; Fraser and Chapple, 2011; Dudareva et al., 2013; Muhlemann et al., 2014; Widhalm and Dudareva, 2015). Some important genes for the biosynthesis of phenylpropanes have been confirmed in petunia (Boatright et al., 2004; Moerkercke et al., 2009).

*Prunus mume* (mei) from Rosaceae, a traditional ornamental tree, was domesticated in China more than 3000 years ago (Chen, 1996). Numerous ornamental cultivars with colorful flowers, various types of branches and flower, variety of flowering periods, and characteristic floral scents have been bred (Sun et al., 2013; Xu et al., 2014; Zhang et al., 2018; Zhuo et al., 2021). As the only species within the *Prunus* genus to produce a strong floral scent (Zhang et al., 2019b), it is important to study the molecular mechanism behind the biosynthesis in *P. mume*’s floral scent. In previous studies, benzyl acetate and eugenol were revealed to be the main components of the headspace volatiles of the unique fragrances of different *P. mume* cultivars; additionally, benzyl alcohol and cinnamyl acetate were found to be the main components of the floral volatiles of the *P. mume* cultivar ‘Fenhong Zhusha’ (‘FZ’) (Zhang et al., 2019a; Bao et al., 2020). All of these volatiles were shown to be biosynthesized by the phenylpropane biosynthesis pathway (Hao R. et al., 2014; Hao R.-J. et al., 2014; Zhang et al., 2019a). Consistent with the pattern of emission of most floral scents (Fenske et al., 2015), the emission and biosynthesis of benzyl acetate and eugenol peak when *P. mume* is in full bloom (Hao R.-J. et al., 2014; Bao et al., 2019; Zhang et al., 2019b). The intracellular levels of metabolites of floral scent components in flower buds have also been identified in five hybrids of *P. mume* (Bao et al., 2020). The encoding genes, enzymatic activities, and functions of *PmBEATs* and *PmBARs*, which are closely related to the biosynthesis of benzyl acetate, have been determined within the *P. mume* genome (Zhao et al., 2017; Bao et al., 2019; Bao et al., 2020). The gene families *PmEGSs* and *PmCFATs*, which encode key functional enzymes involved in eugenol biosynthesis, have also been identified and characterized (Zhang et al., 2019b). However the epigenetic mechanisms involved in the biosynthesis of its floral scent have rarely been reported.

DNA methylation, one of the best-studied epigenetic modifications, plays a key role in genome stability, developmental regulation, gene expression regulation, transposon silencing, and environmental adaptation (Cubas et al., 1999; Zhu, 2009; Law and Jacobsen, 2010). In plants, DNA methylation mainly occurs on cytosine, including within segments with the sequence “CG,” “CHG,” or “CHH” (with H representing A, T, or C) (Finnegan and Kovac, 2000). The dynamic changes of DNA methylation level (ML) have been shown to regulate the expression of genes and ultimately lead to different phenotypes. The methylation status varies among different species, cultivars of the same species, and even tissues of the same plant (Vaughn et al., 2007; Suzuki and Bird, 2008). Evidence has shown that DNA methylation is involved in regulating the expression of key genes during the ripening of tomato fruit (Zhong et al., 2013); drought resistance (Xu et al., 2018) and flowering (Xing et al., 2019) of apple; peel color formation of apple (Bai et al., 2016; Li et al., 2019), red pear (Wang et al., 2013), and sweet orange (Huang et al., 2019); and the flower color formation of *P. mume* chimera (Jiang et al., 2020). However, the role it plays in the process of floral scent biosynthesis has not yet been reported.

Here, by using transcriptome sequencing (RNA-seq) and whole-genome bisulfite sequencing (WGBS), we discuss the methylation modification associated with floral scent biosynthesis in *P. mume* cv. ‘FZ’. The results provide new genome-wide evidence that deepens our understanding of the epigenetic regulatory mechanism underlying the floral scent trait.

## Materials and Methods

### Plant Materials

**FIGURE 1:**
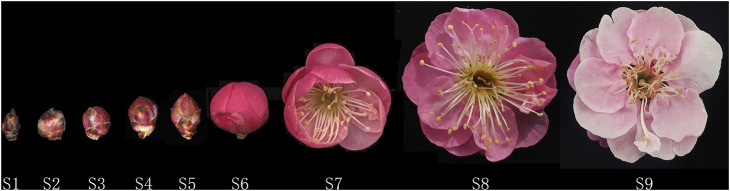
Definition of different flowering stages of *P. mume*: bud scale tight packed (S1), bud swelling (S2), sepal tips appeared (S3), sepals clearly visible (S4), petals appeared (S5), petals clearly visible (S6), beginning of blossom (S7), full blooming (S8), and petal drop (S9).

The present study focused on ‘FZ’, the earliest flowering *P. mume* cultivar (Yuan et al., 2020) with a relatively long flowering time and pleasant smell, which was planted in Jiufeng International Mei Garden (40°03′53″N, 116°05′49″E, 132 m a.s.l.), Haidian, Beijing. To reflect the bud developmental state of the whole plant, all buds on one to two twigs of each branch from three different sides of each experimental tree were collected as one biological replicate. Three biological replicates (1, 2, and 3) constituted one sample. Samples were frozen in liquid nitrogen immediately after being obtained and stored at −80°C until sequencing. We selected four samples in total, and took the S2/S4/S6/S8 bud development stage as the dominant stage, from January 9^th^ to March 13^th^ in 2019 (Figure 1). The dominant stages were determined by the following reasons: some of the key floral components of *P.mume* began to evaporate/emit in the early stages of flowering and peak when *P. mume* is in full bloom (S8). Slightly earlier than that, the biosynthesis of eugenol can be extracted before blooming (S6) (Zhang et al., 2019b) and the expression of key enzyme-encoding genes closely related to floral scent biosynthesis (like *PmCFATs* and *PmBEATs*) in the budding phase (S4 or S5) (Bao et al., 2019; Zhang et al., 2019b). The sample (S2) on January 9^th^ 2019 was also selected for sequencing, for considering that the buds of ‘FZ’ were already finished endo-dormancy on December 23^th^ 2018, and the methylation modification may be earlier than the onset of gene expression (Yuan et al., 2020).

### Whole-Genome Bisulfite Sequencing and Analysis

Genomic DNA of all four sample groups was extracted using Plant Genomic DNA Kit DP305 (Tiangen Biotech, Beijing, China) and then used for library construction using Illumina’s standard DNA methylation analysis protocol and the Accel-NGS®Methyl-Seq DNA Library Kit. The sequencing process was performed on the Illumina Hiseq 6000 platform to generate raw reads, which were preprocessed with Trimmomatic v0.36 software and FastQC analysis (Langmead and Salzberg, 2012). Finally, the clean reads were obtained for subsequent analysis.

### RNA Isolation and Sequencing

Total RNA was extracted with the DP422 extraction kit (Tiangen Biotech). RNA quality and quantity were determined using 1% agarose gels and a NanoPhotometer spectrophotometer (Implen, Calabasas, CA, United States), respectively. An RNA Nano 6000 Assay kit of the Bioanalyzer 2100 system (Agilent, Carpinteria, CA, United States) was used to assess RNA integrity. A total of 3 μg of RNA was used as the input material for sequencing library construction. The NEBNext Ultra RNA Library Prep Kit for Illumina (NEB, Ipswich, MA, United States) was used for reverse transcription. The libraries were also sequenced on the Illumina Hiseq 6000 platform to generate 150 bp paired-end reads (clean bases > 9G).

### Sequence Mapping

Using the HISAT2 (https://daehwankimlab.github.io/hisat2/) and Bismark (https://github.com/FelixKrueger/Bismark.git) software to aligned the clean reads in transcriptome and methylome (Kim et al., 2015; Krueger and Andrews, 2011; Langmead and Salzberg, 2012; Wang et al., 2014) to the reference genome of *P. mume* (http://prunusmumegenome.bjfu.edu.cn/) (Zhang et al., 2012), respectivly. The sequencing depth and coverage were estimated according to duplicates that aligned to a unique genomic region (Krueger and Andrews, 2011; Wang et al., 2014). The non-conversion rate (r) of bisulfite treatment was defined as the rate of the number of sequenced cytosines at all cytosine reference positions divided by the number in the lambda genome.

### Methylated Cytosine Site Analysis and Detection of Differentially Methylated Regions

mC sites were detected using Bismark (Krueger and Andrews, 2011) and defined using a binomial test, with thresholds of sequence depth ≥5 and q-value ≤ 0.01 (Lister et al., 2009; Gifford et al., 2013; Habibi et al., 2013). ML of C site was calculated as follows: ML = mC/(mC + umC) (C here indicate one single C site). The “mCG,” “mCHG,” and “mCHH” contexts (methylcytosine occurring at CG, CHG, and CHH regions, respectively) and their densities, as well as their distributions in each chromosome, were analyzed using previous methods (Chan et al., 2005; Krzywinski et al., 2009; Lister et al., 2009; Zhong et al., 2013). Between samples, the global ML and distributions were also tested (Song et al., 2013). DMRs and differentially methylated loci were identified using DSS software (Feng et al., 2014; Wu et al., 2015; Park and Wu, 2016). Among chomosomes, every 200bp were grouped in one bin. In different functional region of genes, each region is evenly divided into 50 bins. In genesbody and its up- and downstream, each region is evenly divided into 20 bins. The average ML (ML_avg_) and methylation density (MD) of each bin was calculated as follows: MD = mCX/CX (coverage > 5X, C here indicate all C site within each bin, CX means CG, CHG or CHH) (Wang et al., 2015).

$$ML_{avg}=\frac{\sum_{i=1}^{n}ML_{i}}{n}$$

### Differentially Methylated Gene (DMG) Detection and Differentially Expressed Gene (DEG) Analysis

Differentially methylated genes were defined as genes with DMRs within 2 kb of their promoter or gene body (from TSS to TES) region. To clarify the possible regulatory effects of methylation in the natural flowering process of *P. mume*, the differentially expressed genes (DEGs) between two samples were determined using the following criteria: false discovery rate (FDR) < 0.05 and |fold change| ≥0.

### Functional Annotation of DEGs and DMR-Related Genes

The GOseq R soft package was used for Gene Ontology (GO) enrichment analysis of DMR-related genes that were aligned with GO (p-value < 0.05) (Young et al., 2010). Kyoto Encyclopedia of Genes and Genomes (KEGG) analysis was used to explore pathways of DMR-associated genes (Kanehisa et al., 2008). We also used KOBAS software to test DMR-related genes’ statistical enrichment in the KEGG pathways (Mao et al., 2005).

### Methylation in Key Pathways in the Biosynthesis of Floral Fragrance

To show the involvement of methylation in the biosynthesis of *P. mume*’s flower scent, DMGs enriched in eight pathways that are related to floral scent biosynthesis were listed. We then summarized and drew the key pathway of floral scent biosynthesis, phenylpropanoid biosynthesis, and marked the processes involving DMGs with red asterisks. For further information about the methylation involved in regulating the expression of floral scent-related genes, the FPKM (Fragments Per Kilobase of exon model per Million mapped fragments) values of 145 summarized genes in previous reaserch that encoding the key enzymes in the biosynthesis of the dominant floral volatile of *P. mume* were used to make heat maps using the heatmap option and the normalized FPKM values of genes (row) in TBtools software. The DMGs were marked and classified according to region (promoter, gene body), context (CG, CHG, CHH), and ML change (hyper, hypo) properties on heatmap.

### Quantitative Real-Time-PCR Validation

The qRT-PCR was performed on the CFX96 TouchTM Real-Time PCR Detection System (Bio-Rad, Hercules, California, United States) using the following parameters: 95°C for 5 min, 30 cycles of 95°C for 5 s, and 60°C for 30 s, concluding with a melting-curve stage for 10 s at 95°C, 5s at 65°C, and 5 s at 95°C. Each reaction consisted of 0.8 μL 1^st^ strand cDNA, 10 μL SYBR Premix Ex Taq (Takara, Dalian, Japan), 0.8 μL each of 10 mM primer pairs, and 7.6 μL H_2_O. Each sample was assessed in three biological replicates for each sample and normalized using PmPP2A as an internal control. The transcription levels were determined using the 2^-△△Ct^ method. The correlation coefficients between RNA-seq and qRT-PCR were calculated with the CORREL function in an Excel spreadsheet. The specific primers were designed online by Primer 3 plus (http://www.primer3plus.com/cgi-bin/dev/primer3plus.cgi) and are listed in Supplementary Table S9.

## Results

### DNA Methylation Landscape in *Prunus mume* cv. ‘FZ’

To investigate the modification of the biosynthesis of floral scent by DNA methylation during the natural flowering process of mei, we performed WGBS of *P. mume* buds at four different flowering stages: S2, S4, S6, and S8 (hereafter referred to as Fm1–Fm4) (Figure 1). Each stage was sequenced with three biological replicates. For each sample, at least 32 million clean reads were produced (Supplementary Table S2). Approximately 60% of the clean reads were mapped to the reference genome using Bismark (Krueger and Andrews, 2011) (Supplementary Table S2). The average genome coverage was 21.67 × (Supplementary Table S3). All of our sequenced methylated regions had ∼21 × average coverage per chromosome and ∼10 × average coverage per cytosine site, covering an average of 83.8% of the genome (Supplementary Figure S1; Supplementary Table S4).

**FIGURE 2:**
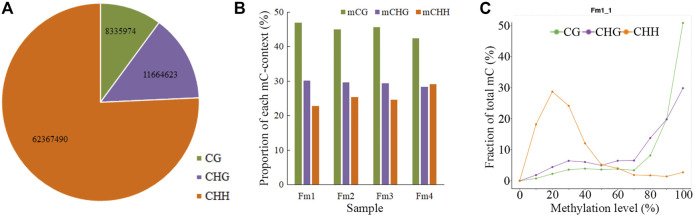
Proportions of the three different contexts (CG, CHG, CHH) in **(A)** total cytosine **(C)** sites, **(B)** total mC (methylated cytosine) sites, and (C) ML distribution. The mC distribution diagrams of all samples are shown in the Supporting Information (Figure S4).

Among the 82,368,087 C (cytosine) sites in the *P. mume* genome, the CHH context was the most common and the CG context was the least (Figure 2A). In each sample, ∼8% (7.32–8.46%) of the total C sites were methylated (Table 1). The mC sites mainly featured the mCG context (42.44–46.94%), with the mCHG (28.40–30.19%) and mCHH contexts (22.86–29.14%) comprising the rest (Figure 2B). Among the three contexts of C sites, 33.90%–35.72% of CG regions, 15.60%–16.97% of CHG regions, and 2.21–3.26% of CHH regions were methylated (Table 1). Meanwhile, it was shown that chromosome Pm3 was highly methylated (Supplementary Figure S2). In terms of ML, most mCG and mCHG regions had higher ML (80–100%), while most mCHH regions showed lower ML (10–40%) (Figure 2C). The analysis of methylation within chromosomes showed that both high ML_avg_ and MD areas were enriched in pericentromeric regions, where the gene density is low (Supplementary Figure S3). The results are consistent with findings from the petals of *P. mume* cv. ‘FT’ (Jiang et al., 2020), apples, and *Arabidopsis*.

**TABLE 1:** Proportion of mC in the three different contexts (CG, CHG, CHH) of four different flowering stages of *Prunus mume* ‘Fenhong Zhusha’ whole genome.

Samples	mC	mC/C	mCG	mCG/CG	mCHG	mCHG/CHG	mCHH	mCHH/CHH
Fm1	6032102	7.32%	2826502	33.90%	1820358	15.60%	1385241	2.21%
Fm2	6628481	8.04%	2978181	35.72%	1960418	16.80%	1689881	2.70%
Fm3	6226425	7.55%	2841509	34.08%	1852006	15.87%	1532909	2.45%
Fm4	6977067	8.46%	2958821	35.49%	1980533	16.97%	2037713	3.26%

### Methylation Level Distribution During Flowering of *Prunus mume* Buds

**FIGURE 3:**
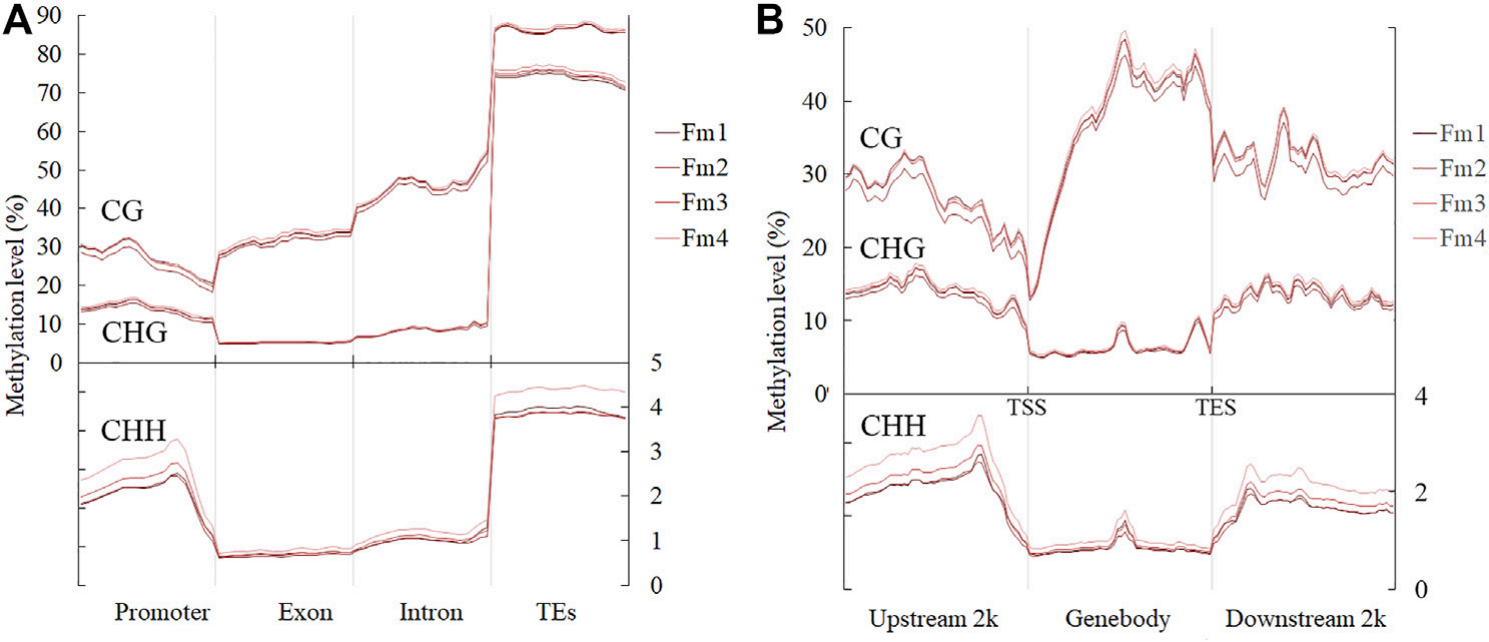
ML in **(A)** different gene functional regions and **(B)** gene body and regions 2 kb up- and downstream of it, during flowering. TSS and TES stand for transcription start and end sites, respectively.

The average values of ML in the mCG, mCHG, and mCHH contexts were 38.13% (36.29–39.32%), 17.25% (16.47–17.96%), and 1.85% (1.69–2.15%), respectively, while the average global ML in flower buds of *P. mume* was 8.35% during the flowering process (Table 2). Among different gene functional regions, all CX contexts had the highest ML in the TE region (Figure 3A). Among the gene body and 2 kb regions up- and downstream of it, the CG context had higher ML in the gene body, while the CHG and CHH contexts had lower ML in the gene body than in other regions (Figure 3B).

**TABLE 2:** Global mean ML of mC in the three different contexts (CG, CHG, CHH) of different flowering stages of *Prunus mume* ‘Fenhong Zhusha’.

Samples	Mean mC(%)	Mean mCG(%)	Mean mCHG(%)	Mean mCHH(%)
Fm1	8.35	38.51	17.19	1.72
Fm2	7.93	36.29	16.47	1.69
Fm3	8.38	38.42	17.39	1.83
Fm4	8.75	39.32	17.96	2.15

**FIGURE 4:**
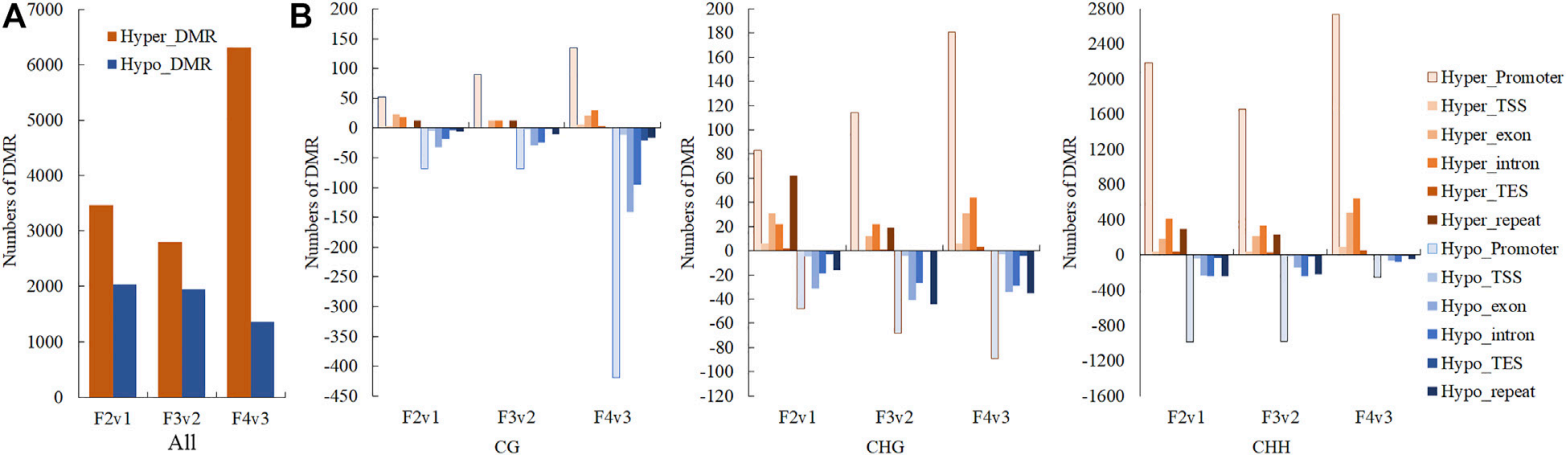
Numbers of hyper- and hypo-DMRs during flowering, shown in **(A)** all mC site and **(B)** three different contexts (CG, CHG, CHH) and different gene region (promoter, TSS, exon, intron, TES and repeat). The terms “hyper-” and “hypo-” represent DMRs that were hypermethylated and hypomethylated, respectively.

In the search for DMR-related genes, we detected 5492 (3461 hyper- and hypomethylated), 4744 (2796 hyper- and 1948 hypomethylated), and 7677 (6320 hyper- and 1357 hypomethylated) DMRs within three methylome comparisons between F2v1, F3v2, and F4v3, respectively. During the flowering process, the number of hyper-DMRs was greater than that of hypo-DMRs (Figure 4A). As shown in this Figure 4B, the number of DMRs was mainly from the CHH context, which occurs in promoter regions, and the number of hyper-DMRs was larger than that of hypo-DMRs. The result is consistent with the gradual increase of ML in promoter regions in the CHH context during flowering (Figures 3A,B). In contrast, the number of hypo-DMRs in the final stage of flowering was mainly contributed to by hypomethylated CG-DMRs in promoter regions (Figure 4B).

### DMGs Participate in the Pathway of Floral Scent Biosynthesis

**FIGURE 5:**
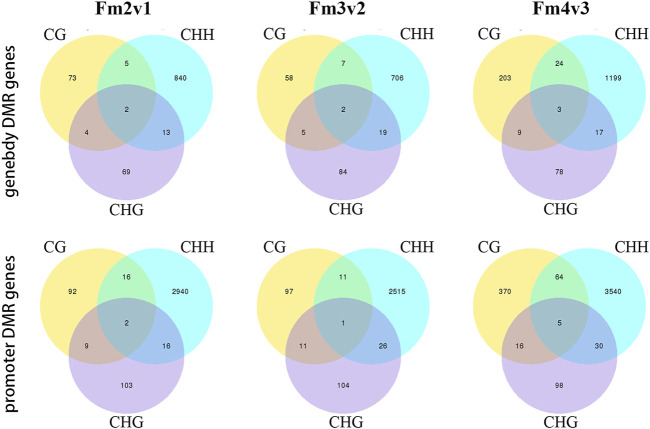
Numbers of genes related to DMRs within promoters and gene bodies, shown for three contexts.

Next, we detected 4005 (promoter, 3178; gene body, 1006), 3515 (promoter, 2765; gene body, 881), and 5306 (promoter, 4123; gene body, 1533) DMGs, which were associated with DMRs, in comparisons of Fm2v1, Fm3v2 and Fm4v3 (“m” here indicate methylome). In brief, the results showed that the genes related to CHH context DMR within promoter region constituted the largest proportion of DMGs among all methylome comparisons (Figure 5), which is consistent with previous results (Supplementary Table S5). We revealed the functions of those DMGs by KEGG enrichment analysis. Remarkably, some crucial DMGs were enriched in eight KEGG pathways related to floral scent biosynthesis. Overall, 97 (66 promoter, 44 gene body) and 17 DMGs were enriched in phenylpropanoid biosynthesis (ko pmum00940) and its upstream pathway phenylalanine, tyrosine, and tryptophan biosynthesis (ko pmum00400), respectively. Moreover, 20, 11, 14, 4 and 45 DMGs were enriched in the pathways of terpenoid backbone biosynthesis (ko pmum00900), monoterpenoid biosynthesis (ko pmum00902), diterpenoid biosynthesis (ko pmum00904), sesquiterpenoid and triterpenoid biosynthesis (ko pmum00909), and phenylalanine metabolism (ko pmum00360). Furthermore, 371 DMGs were enriched in the biosynthesis of secondary metabolites (ko pmum01110) (Supplementary Table S5).

### Correlation Between DNA Methylation and Gene Expression Levels

Using the same samples of mei buds, we also performed RNA-seq to investigate the DEGs involved in floral scent biosynthesis during the natural flowering process. For each sample, at least 50 million (average 62 million, 9.32G over 30× coverage) clean reads were produced (Supplementary Table S6). Over 91% of the clean reads were mapped to the reference genome using Bismark (Supplementary Table S6) (Krueger and Andrews, 2011).

**FIGURE 6:**
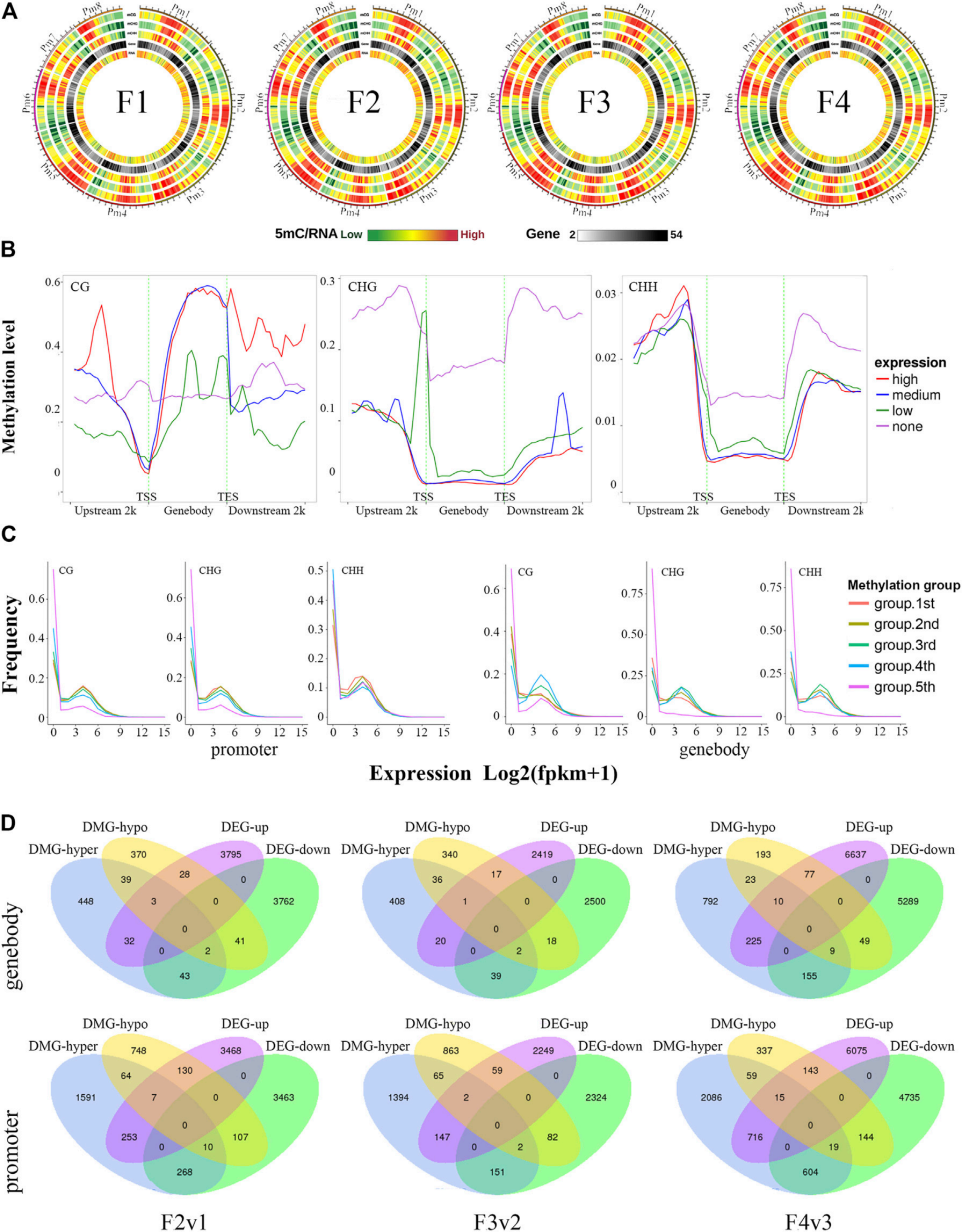
Correlation between ML and expression level of genes, shown in three different CX contexts. **(A)** The overall relationship between ML and expression level on chromosomes. **(B)** The MLs of gene body and regions 2 kb up- and downstream of it in different CX contexts of genes under different expression levels. FPKM_25% and FPKM_75% refer to values at the boundary of the 25th and 75th percentiles of expression levels, respectively. Each region of each gene was divided equally into 50 bins, and the MLs of all CX sites in each bin were averaged as the ML of the bin. **(C)** The expression level distribution frequencies of six groups of genes with different MLs from low to high, shown separately for gene body and promoter regions. DNA methylation levels were classified into five groups: group 1 (red; 0 < methylation level < level_20%); group 2 (yellow-green; level_20% ≤ methylation level < level_40%); group 3 (green; level_40% ≤ methylation level < level_60%); group 4 (turquoise; level_60% ≤ methylation level < level_80%); and group 5 (blue; methylation level ≥ level_80%). Level_20%, _40%, _60%, and _80% represent values at the boundaries of the 20th, 40th, 60th, and 80th percentiles of methylation levels. **(D)** Venn plots showing the numbers of DEGs that were up- or downregulated in each comparison. The terms “hyper-” and “hypo-” represent DMRs within DEGs that were hypermethylated and hypomethylated, respectively. The results are displayed according to DMRs within promoter or gene body regions.

At the chromosome level, low ML and high gene expression levels were distributed in the same region on the chromosome. Higher gene density was also observed in this region (Figure 6A). All genes were divided into four groups according to their expression levels from low to high, and their average ML was determined in different gene regions (gene body and 2 kb up- and downstream of it, TSS, and TES). Among all methylated genes (MGs) at each flowering stage, a higher expression level of the gene was associated with a lower methylation level of the TSS region. Furthermore, higher average ML was detected in the promoter regions of the CG and CHG contexts of unexpressed genes (Figure 6B).

The MGs were divided into five groups (groups 1–5) according to their ML from low to high, and the gene distribution frequencies (y-axis) at different expression levels (x-axis) were represented graphically for each group. In the promoter regions of the CG- and CHG-context MGs, a higher ML was associated with more genes being distributed in the low-expression region log2(FPKM+1) <1, and fewer genes being distributed in 2 < log2(FPKM+1) < 8. The CHH-context MGs also had similar distributions in frequency, although genes with the highest ML (group 5) appeared to have more genes in region 2 < log2(FPKM+1) < 8 than group 4 (Figure 6C). The results indicated that ML in the promoter regions was negatively correlated with expression level. In the gene body regions, the MG group with the highest ML was dominated by a low expression level in all CX contexts. From groups 1 to 3, a higher ML was associated with a higher frequency of genes in the expression level of region 2 < log2(FPKM+1) < 8. The results indicated that ML in gene body regions is positively correlated with expression level (Figure 6C).

The comparison of F2v1 in Figure 6D is here taken as an example. In the gene body region, there were 32 upregulated and 43 downregulated genes in hyper-DMG, and 28 upregulated and 41 downregulated genes in hypo-DMG. Meanwhile, in the promoter region, there were 253 upregulated and 268 downregulated genes in hyper-DMG, and 130 upregulated and 107 downregulated genes in hypo-DMG. Similar results were also found in F3v2 and F4v3. Some of the DMGs have both hyper- and hypomethylated regions (Figure 6D). These results showed that, when limiting the analysis to certain genes, the expression levels of many genes do not always conform to having a negative correlation with the ML in the promoter and a positive correlation with the ML in the gene body.

### DMR-Associated DEGs Widely Involved in Floral Scent Biosynthesis

We detected 7706 (upregulated 3848; downregulated, 3858), 5016 (upregulated 2259; downregulated, 2457), and 12,451 (upregulated 5502; downregulated, 6949) DEGs in compairsion Fr2v1, Fr3v2 and Fr4v3, respectively (“r” here indicate transcriptome) (Figure 6D). The results of KEGG enrichment analysis showed that a total of 214 DEGs were involved in seven floral scent biosynthesis pathways. As the key pathway for biosynthesis of the characteristic aroma of *P. mume*, phenylpropanoid biosynthesis (ko pmum00940) was enriched the most for DEGs (96 DEGs). Meanwhile, its upstream pathway, phenylalanine, tyrosine, and tryptophan biosynthesis (ko pmum00400), featured 32 DEGs. Moreover, 21, 39, 13, 14, and 10 DEGs were involved in phenylalanine metabolism (ko pmum00360), terpenoid backbone biosynthesis (ko pmum00900), monoterpenoid biosynthesis (ko pmum00902), diterpenoid biosynthesis (ko pmum00904), and sesquiterpenoid and triterpenoid biosynthesis (ko pmum00909), respectively (Supplementary Table S7).

**FIGURE 7:**
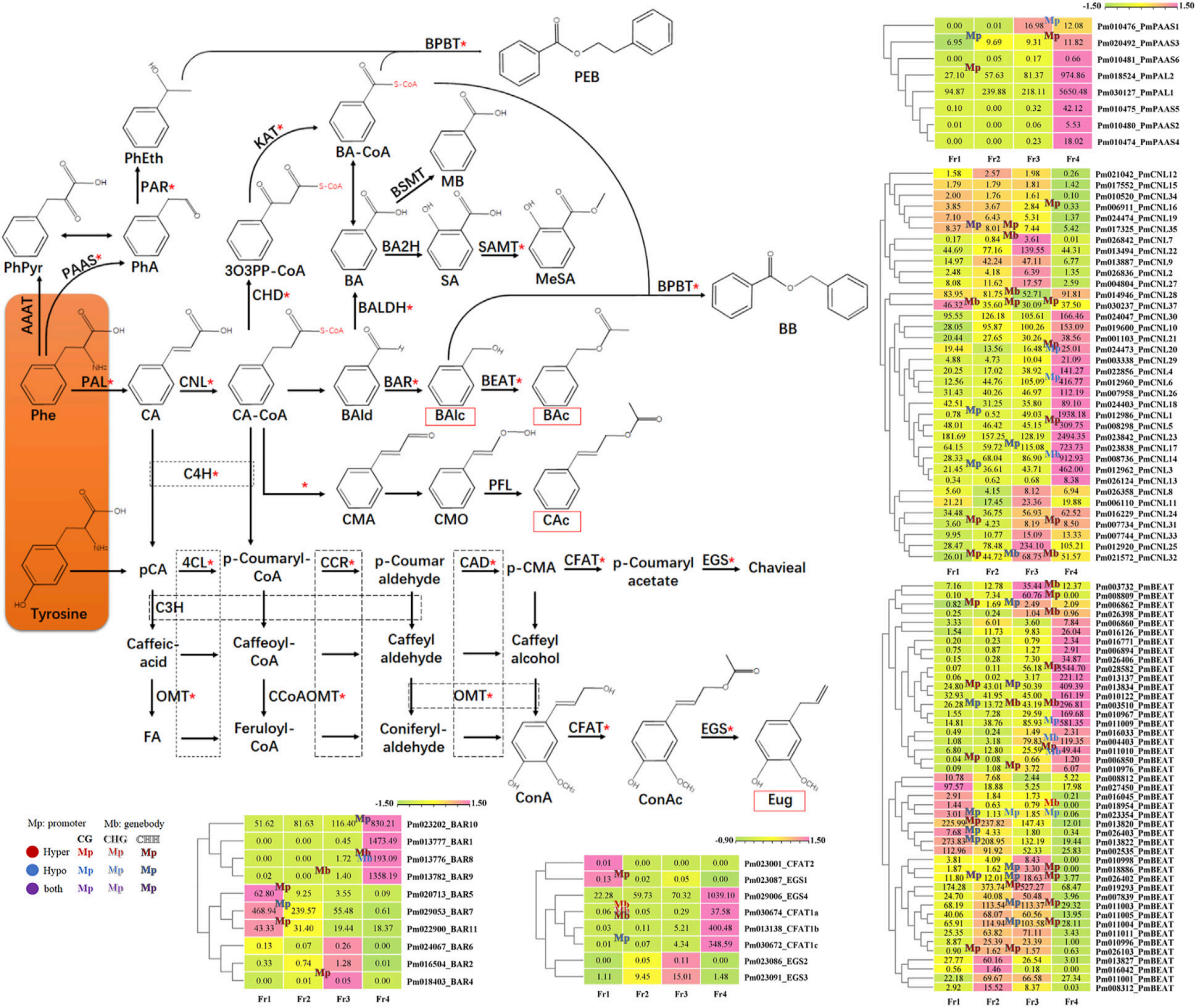
Methylation modification and the heat maps of DEGs encoding key enzymes in the pathway of floral scent biosynthesis in *P. mume*. The key floral scent biosynthetic pathway in *P. mume* is shown. The biological compounds on an orange background are the starting substrates of the phenylpropane pathway, synthesized by the upstream pathway. The red rectangles indicate the metabolites that were detected in the headspace. Red asterisks indicate that genes encoding enzymes have undergone differential methylation during the process. The dashed boxes indicate that the processes involved share the same enzyme. In the heat maps, pink and light-green represent high and low transcript expression, respectively. The methylation statement were also marked in the heat maps with “Mp” and “Mb” to indicate the differential methylation occurred in promoter and genebody region, respectively; Text color with red, blue and purple represent ML up (hypermethylated), down (hypomethylated) and both happened respectively; Different fonts refer to different CX context, as shown in the figure.

Over 90% of the volatile components of *P. mume* fragrance were shown to be synthesized by the phenylpropane biosynthetic pathway. We also integrated some genes that previously characterized in the biosynthesis of other characteristic fragrance components in *P. mume* into the phenylpropane biosynthetic pathway, and then marked the processes involving DMGs with red asterisks (Figure 7). The results clearly showed that DMGs were widely involved in almost all steps in the phenylpropanoid biosynthesis process, including not only the dominant and key components, but also the low (FPKM value < lower quartile) or none (FPKM value < 1) part in *P. mume* fragrance.

We further summarized 145 genes that were related to the key enzyme in the phenylpropane biosynthetic pathway, as identified in our published results of *P. mume* floral fragrance research (Bao et al., 2019; Bao et al., 2020; Zhang et al., 2019b; Zhao et al., 2017), including 2 *PmPALs*, 6 *PmPAASs*, 37 *PmCNLs*, 11 *PmBARs*, 81 *PmBEATs*, 4 *PmCFATs*, and 4 *PmEGSs*. According to the heatmap results, among 47 candidate genes that exhibited an expression pattern matching the pattern of floral scent emission, 22 were differentially methylated during the flowering process (Figure 7, Supplementary Table S10). Some of these genes were proven to encode key enzymes involved in the characteristic fragrance of *P. mume* in previous studies, such as *PmCFAT1a/1c* (Zhang et al., 2019b), *PmBEAT36/37* (Bao et al., 2019), and *PmBAR8/9/10* (Bao et al., 2020); or to have the same expression pattern as in other *P. mume* cultivars, such as *PmPAL2*, *PmPAAS3*, and *PmCNL1/3/5/6/14/17/20* (Zhao et al., 2017; Bao et al., 2020) (Supplementary Table S8).

Among them, *PmCFAT1a*, which have been proved to produced eugenol through an enzyme assay (Zhang et al., 2019b), had four hyper-DMR in its genebody region during F1 to F2, involving all three CX contextsthe with ML changes from: 26.19–44.65% (CG), 20.05–39.34% (CHG), 2.43–5.64% and 1.06–3.45% (CHG). The ML of all hyper-DMR exceeded the average ML of the sample (Table 2; Figure 7). Pm011009 and Pm011010 (*PmBEAT36* and *PmBEAT37*) have been reported to significantly positive affect the synthesis of benzyl acetate in the petal cells of *P. mume* when overexpressed (Bao et al., 2019). Pm011009 had CG context hypo-DMR (90.49–81.02%) in the promoter region while the gene expressing were significantly increased in F3 to F4. Pm011010, which have been reported to inhibited the expression of Pm011009 in protoplasts, had CHH context hyper-DMR (22.41–39.87%) in its promoter region and CG context hypo-DMR (41.82–17.63%) in its genebody region from F3 to F4, although the expressing were also increased (Figure 7). We randomly selected 6 genes to do qRT-PCR to verify the transcriptome results. The qRT-PCR results showed a similar trend to the FPKM value (Supplementary Figure S4), proving that the transcriptome results are reliable.

## Discussion

The modification of DNA sequences by methylation is one of the heritable types of gene expression that does not change the genetic background. Research on DNA methylation thus has advantages in explaining the different phenotypes caused by differential gene expression in the same or similar genetic backgrounds. Studies have also shown variations in floral scent among different *P. mume* cultivars, despite strong similarity in their genetic background. As the only species of *Prunus* that produces a floral fragrance (Zhang et al., 2019b), research on the molecular mechanism regulating floral scent biosynthesis in *P. mume* is important.

Our results showed that DMGs were widely involved in eight recognized floral scent biosynthesis pathways during the natural flowering process of *P. mume*. Among these, the phenylpropane biosynthesis pathway was found to have the most enriched DMGs, which is consistent with the fact that over 90% of the floral volatiles emitted by the *P. mume* cv. ‘FZ’ are synthesized by the phenylpropane biosynthesis pathway (Zhang et al., 2019a). These DMGs participate in most of the floral biosynthetic processes in the phenylpropane biosynthetic pathway. All of these results indicated that methylation plays a role in the process of floral scent biosynthesis.

We selected 47 candidate genes that exhibit an expression pattern matching the emission of floral scent, 22 of which were differentially methylated during flowering. Some of these genes have been proven to encode key enzymes involved in the characteristic floral scent of *P. mume* in previous studies, such as *PmCFAT1a*/*1c* (Zhang et al., 2019b) and *PmBEAT36*/*37* (Bao et al., 2019), or have the same expression pattern in other *P. mume* cultivars, such as *PmPAL2*, *PmPAAS3*, *PmBAR8*/*9*/*10*, and *PmCNL1*/*3*/*5*/*6*/*14*/*17*/*20* (Zhao et al., 2017; Bao et al., 2020). These lines of evidence indicate that the ML of DNA is involved in regulating the expression of genes encoding key enzymes in floral scent biosynthesis, and ultimately forms the unique floral scent of *P. mume*.

Notably, the DEGs involved in floral scent biosynthesis showed diverse patterns of methylation. Although our results were consistent with previous studies, hypermethylated regions were usually associated with segments showing low gene expression on chromosomes; and the gene expression level was found to be negatively and positively correlated with the ML of the promoter and gene body regions, respectively, among all methylated genes. However, when limiting the analysis to certain genes, the relationship between ML and gene expression did not always follow this rule. Similar result can be found in the result of previous research (Ma et al., 2018; Jiang et al., 2020). A more likely explanation was the complexity of the process of floral scent biosynthesis, so that methylation is not the only regulation factor. For example, the promoter of *PmBEAT36*/*37* has elements that respond to temperature and light (Bao et al., 2019). In the future, more research should be carried out within DMRs of key genes involved in floral scent biosynthesis that exhibit expression patterns matching the patterns of volatile emission and that differ in their expression patterns among *P. mume* cultivars with different scents.

In conclusion, our results revealed that DNA methylation is widely involved in the process of floral scent biosynthesis and may play critical roles in regulating such biosynthesis in *P. mume*. This was revealed by a comprehensive analysis of the RNA-seq and WGBS data of flower buds at four different flowering stages of the *P. mume* cv. ‘FZ’. The results provide a new epigenetic perspective that deepens our understanding of the mechanism behind the biosynthesis of floral scent.

## Data Availability

The original contributions presented in the study are publicly available in NCBI under accession numbers PRJNA765446 and PRJNA766216.

## Supplementary Material

Supplementary Table S10 MR related to the 22 candidate genes.

## Glossary

3O3PP-CoA: 3-oxo-3-phenylpropionyl-CoA
4CL: 4-coumarate CoA-ligase
BA: benzoic acid
BAc: Benzyl acetate
BA-CoA: benzoyl-CoA
BAlc: benzylalcohol
BAld: benzaldehyde
BALDH: benzaldehyde dehydrogenase
BB: benzylbenzoate
BEAT: acetyl-CoA:benzylalcohol acetyltransferase
BPBT: benzoyl-CoA:benzylalcohol/2-phenylethanol benzoyltransferase
BSMT: benzoic acid/salicylic acid carboxyl methyltransferase
C4H: cinnamic acid 4-hydroxylase
CA: cinnamic acid
CAc: Cinnamyl acetate
CA-CoA: cinnamoyl-CoA
CAld: Cinnamyl dehyde
CCoAOMT: caffeoyl-CoA O-methyltransferase
CFAT: coniferylalcohol acetyltransferase
CHD: cinnamoyl-CoA hydratase/dehydrogenase
CMA: cinnamaldehyde
CMO: cinnamyl alcohol
CNL: cinnamoyl-CoA ligase
ConA: coniferyl alcohol
EGS: eugenol synthase
Eug: eugenol
FA: ferulic acid
KAT: 3-ketoacyl-CoA thiolase
MB: methylbenzoate
MeSA: Methyl Salicylate
PAAS: phenylacetaldehyde synthase
PAL: phenylalanine ammonia-lyase
PAR: phenylacetaldehyde reductase
pCA: p-Coumaric acid
p-CMA: p-Coumaryl alcohol
PhA: phenylacetaldehyde
Phe: L-phenylalanine
PhEth: 2-phenylethanol
PhPyr: phenylpyruvic acid
SA: Salicylate
Tyr: Tyrosine.
