# Supplementary material for: Integration of Transcriptome and Methylome Analyses Provides Insight Into the Pathway of Floral Scent Biosynthesis in Prunus mume
Source: Front Genet. 2021 Dec 15;12:779557. doi: 10.3389/fgene.2021.779557 (PMC8714837; doi:10.3389/fgene.2021.779557)
Supplement: Supplementary file 10 [file DataSheet1.docx]

Supplementary Material

# Supplementary Figures and Tables

## Supplementary Figures


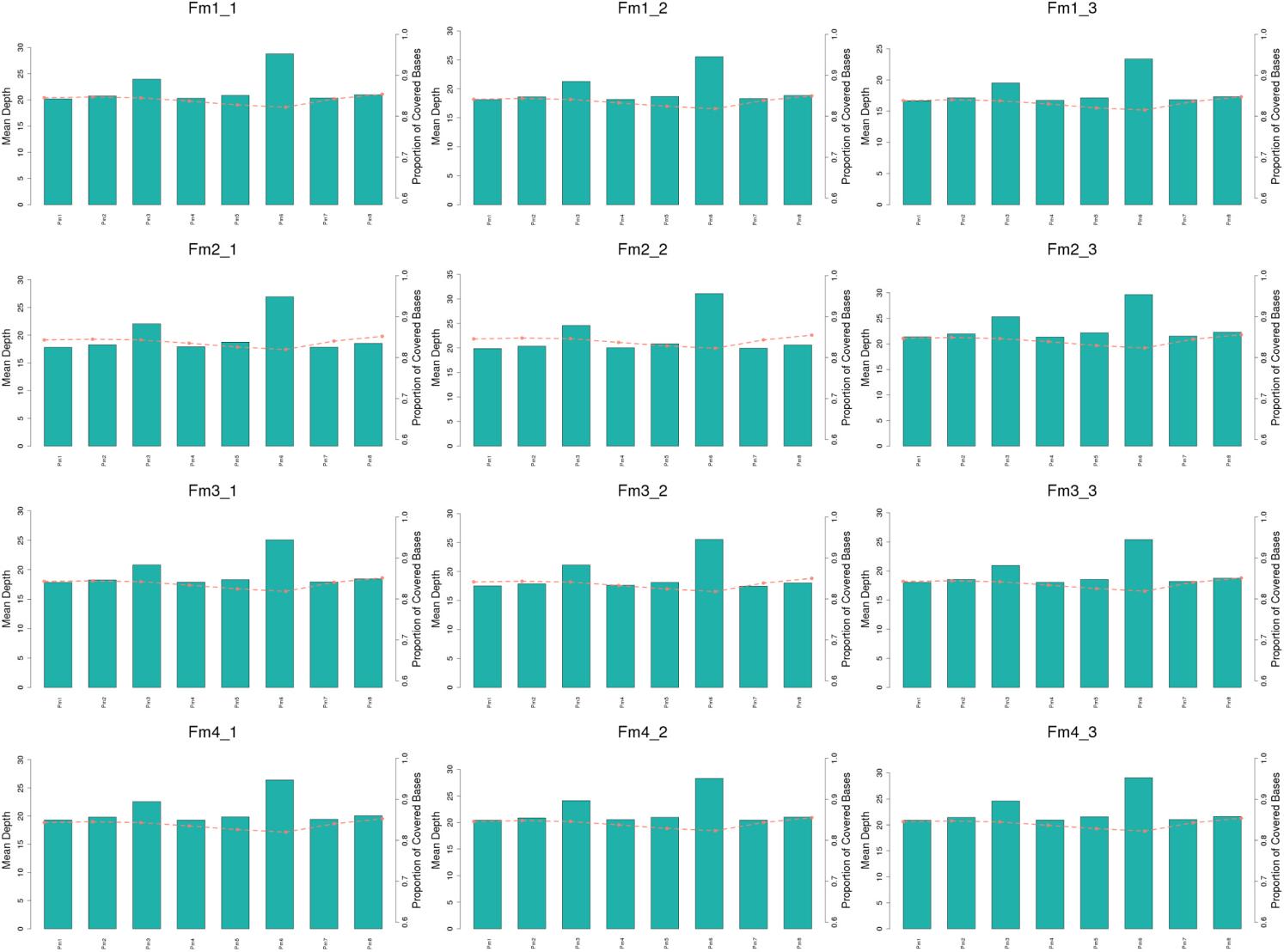


**Figure S1 Distribution of sequencing depth and coverage in chromosome.**

**
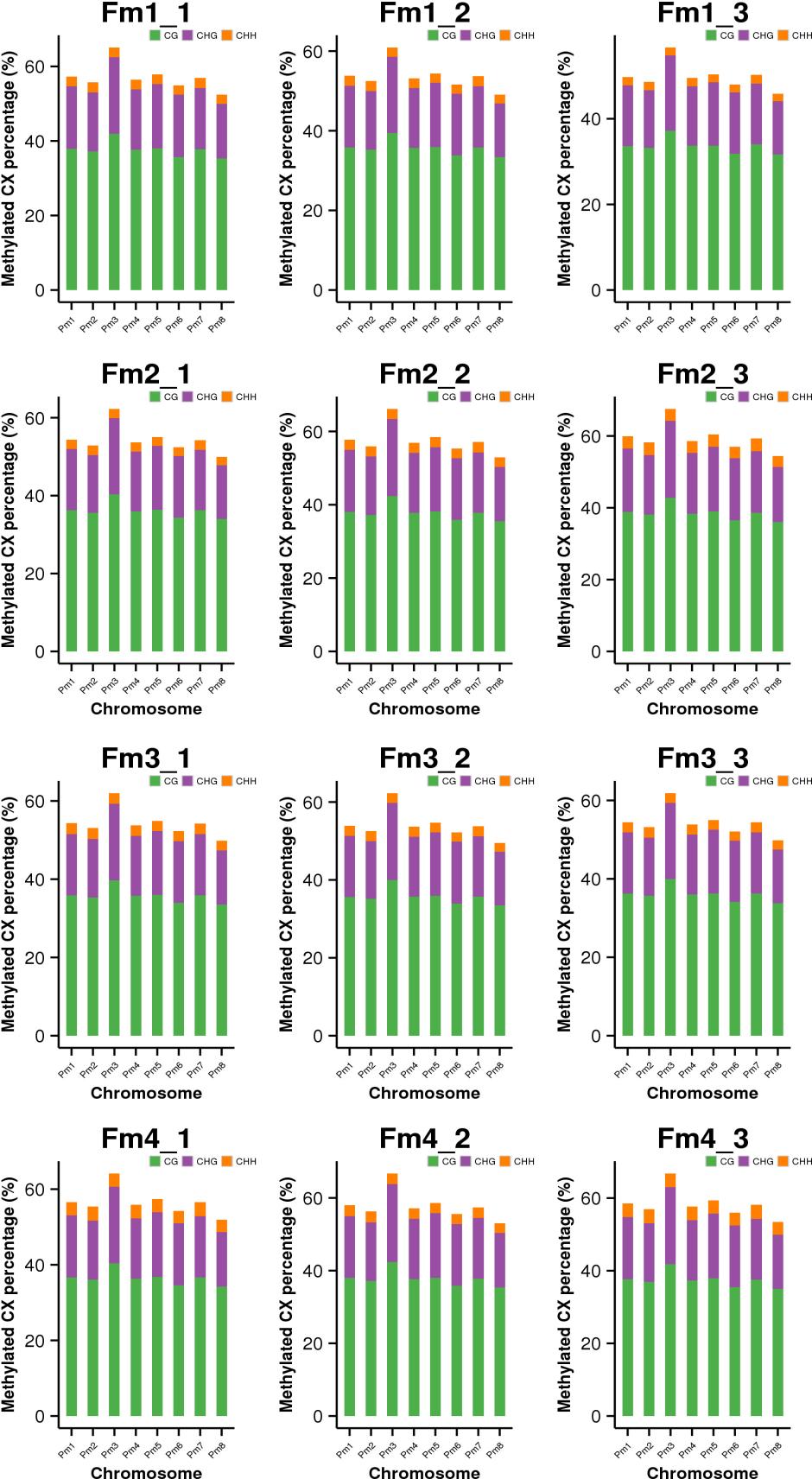
**

**Figure S2** Percentage of mC sites in each chromosome.


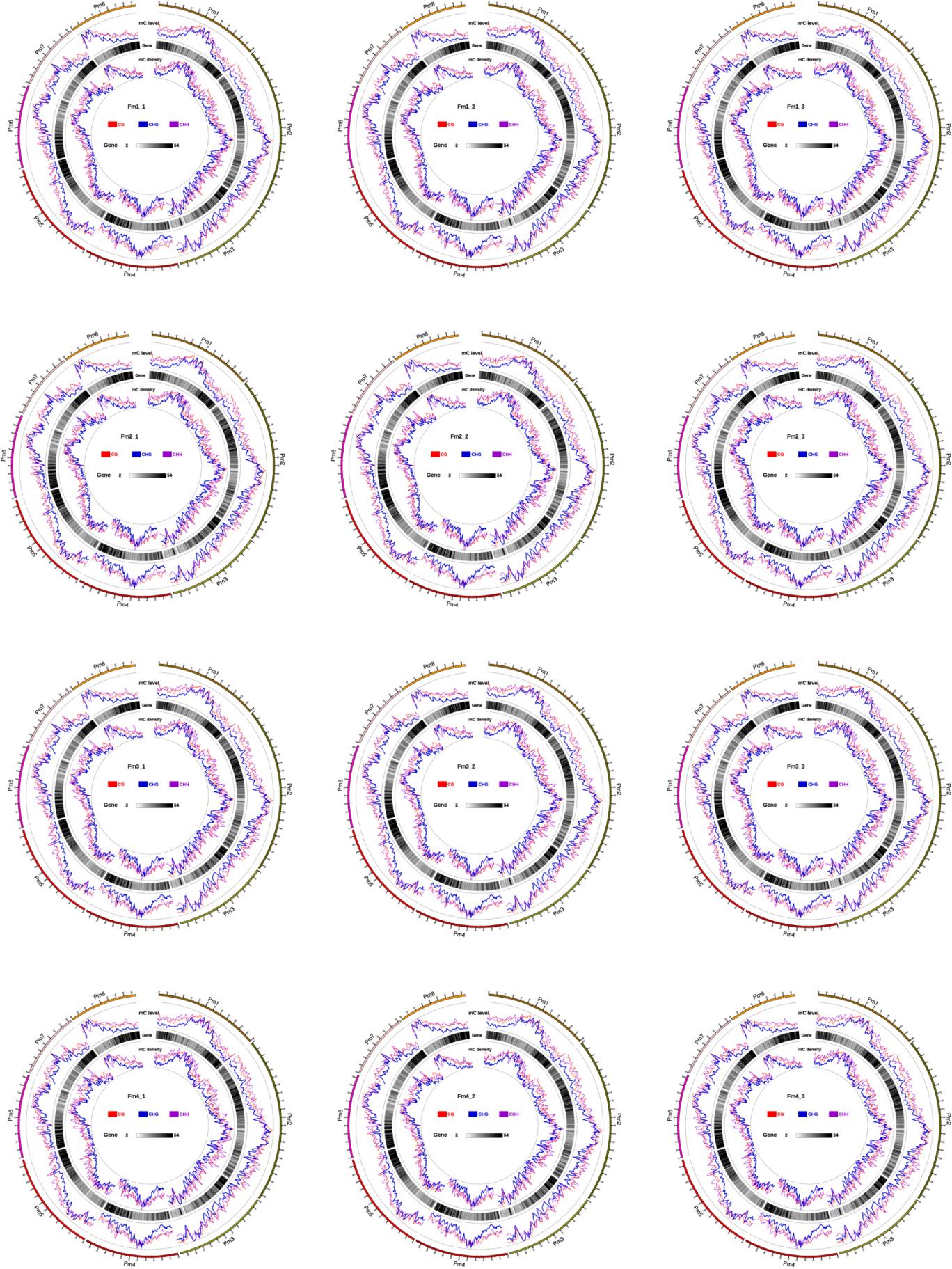


**Figure S3** MD and ML_avg_ distribution within chromosomes.


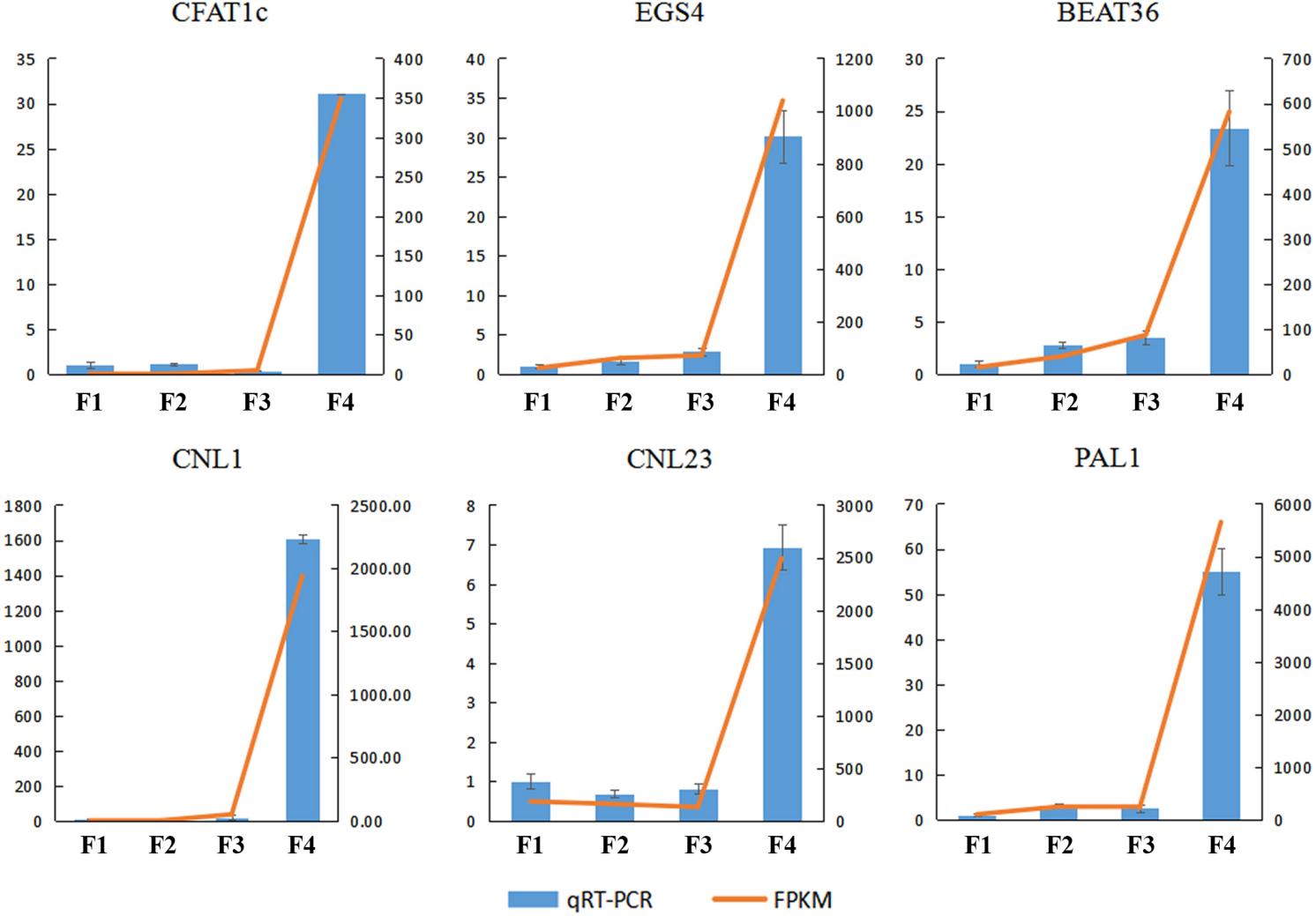


**Figure S4** The qRT-PCR result and FPKM value of genes

## Supplementary Tables

**Table S1** Raw data quality control statistics of methylome.

**Table S2** Mapping summary.

**Table S3** Genome coverage depth statistics.

**Table S4** Summary of cytosines coverage.

**Table S5** KEGG result of eight enriched floral scent related pathway in methylome.

**Table S6** Raw data quality control statistics of transcriptome.

**Table S7** KEGG result of seven enriched floral scent related pathway in transcriptome.

**Table S8** Summarize of 145 genes that were related to the key enzyme in the phenylpropane biosynthetic pathway.

**Table S9** Primer used in qRT-PCR.
